# Optokinetic stimulation for the treatment of vestibular and balance disorders: a systematic review with meta-analysis

**DOI:** 10.1007/s00405-024-08604-1

**Published:** 2024-04-05

**Authors:** Esteban Obrero-Gaitán, Ana Sedeño-Vidal, Ana Belén Peinado-Rubia, Irene Cortés-Pérez, Alfonso Javier Ibáñez-Vera, Rafael Lomas-Vega

**Affiliations:** 1 Department of Health Sciences, University of Jaen, Jaén, Spain; 2 AFIXA Fibromyalgia Association, Jaén, 23008 Spain

**Keywords:** Vestibular diseases, Postural balance, Optokinetic stimulation, Vestibular rehabilitation, Aged

## Abstract

**Objectives:**

To analyse the effectiveness of optokinetic stimulation (OKS) for improving symptoms and function in patients with vestibular and balance disorders.

**Methods:**

PubMed (MEDLINE), SCOPUS, Web of Science (WOS), CINAHL Complete, and PEDro databases were searched to identify randomized controlled trials (RCTs) that included patients with vestibular and balance disorders and compared the effects of OKS versus other interventions or no intervention on subjective or objective functional outcomes. Data were analysed by the standardized mean difference (SMD) and its 95% confidence interval.

**Results:**

A total of 10 studies were selected including 468 patients, 177 of whom received OKS. There were no significant differences in scores on the Dizziness Handicap Inventory (DHI) (SMD = 0.02; 95% CI − 0.18 to 0.23; *p* = 0.83) or the visual analogue scale (VAS) for vertigo (SMD = 0.16; 95% CI − 1.25 to 1.58; *p* = 0.82). However, there were statistically significant differences in the timed up and go (TUG) test, with a large effect (SMD = − 1.13; 95% CI -2 to − 0.28; *p* = 0.009), and in the sensory organization test (SOT), with a medium effect (SMD = − 0.7; 95% CI − 1.21 to − 0.19; *p* = 0.007). Subgroup analysis showed significant effects of OKS on VAS (*p* = 0.017), TUG (*p* = 0.009) and SOT (*p* = 0.001) only in patients with balance disorders without vestibular disease (*p* > 0.05).

**Conclusions:**

OKS may improve dizziness intensity measured with VAS or dynamic balance measured whit TUG and SOT in patients with balance disorders not due to vestibular disease. The quality of the evidence was low or very low due to the small number of included studies.

**PROSPERO Registry number:**

CRD42023445024.

## Introduction

Vestibular and balance disorders have been widely examined since they affect a significant part of the population. In the USA, 35% of adults aged 40 and older have evidence of balance dysfunction [1]. These disorders can cause additional problems, such as increased morbidity among older subjects with multiple comorbidities, who may experience falls and greater use of health resources [2].

Optokinetic stimulation (OKS) consists of exposure to visual large-field motion stimuli [3] that could be used to improve a patient’s tolerance for instability in motion environments. This approach is commonly prescribed to patients with unilateral spatial neglect poststroke [4]. Furthermore, it is increasingly being applied to patients with vestibular disorders, such as dizziness or postural instability while they are in shopping malls or airports; in such environments, many objects are moving at the same time in different directions and at different speeds, thus generating a visually demanding environment [5].

OKS generates visually evoked postural responses that can usually be suppressed by repeated exposure, which indicates learning from ocular and cervical proprioceptors [3] and adaptive neuroplastic changes in visual dependency [6]. In fact, it has been observed that the use of OKS activates cortical areas related to visual motion sensitivity and ocular motor and vestibular function [6].

Some recent research has found that this approach applied through virtual reality reduces visual dependence in postural perception in healthy patients [7]. It has also been suggested that the application of OKS via virtual reality leads to improvements in visual vertigo symptoms in patients with peripheral vestibular dysfunction [8]. Additionally, an earlier study analysed the effects of OKS in 112 patients with unilateral and bilateral vestibular deficits, obtaining normalization of optokinetic nystagmus after 6 to 10 sessions [9].

Based on these findings and considering that, to our knowledge, no systematic review has examined the use of OKS for vestibular disorder rehabilitation, our aim is to carry out a systematic review and meta-analysis to analyse the strongest existing knowledge about the effectiveness of OKS for improving symptoms and function in patients with vestibular and balance disorders.

## Methods

### Study design and protocol registration

This systematic review with meta-analysis was conducted in accordance with the recommendations of the Preferred Reporting Items for Systematic Reviews and Meta-Analyses (PRISMA) statement [10] and the Cochrane Handbook for Systematic Reviews of Interventions [11]. The protocol of this review was registered in PROSPERO with the following registration number: CRD42023445024.

### Literature search and databases

To carry out this systematic review with meta-analysis, the PubMed MEDLINE, SCOPUS, Web of Science (WOS), CINAHL Complete, and PEDro databases were searched up to July 2023. In addition, the reference lists of previously published articles, conference proceedings, expert manuscripts, and grey literature were searched. For the search strategy, we identified two search domains based on the PICOS principle [12]: population, patients with vestibular diseases; and intervention, optokinetic exercises. To develop the search strategy, free terms such as "optokinetic" or "vestibular disorders" were combined with MeSH terms indexed in PubMed MEDLINE. Additionally, the Boolean operators "AND" and "OR" were used to elaborate the search strategy. No filters related to language or date of publication were established. The literature search was carried out by two authors, and a third author provided support in this phase. Table 1 shows the search strategy used in each database.

**Table 1:** Search strategy

Data bases	Search strategy
Pubmed Medline	(Optokinetic [tiab] OR Optokinetic training [tiab] OR Optokinetic response [tiab] OR Optokinetic stimulation [tiab]) AND (Vestibular disease [mh] OR Vestibular disease* [tiab] OR Vestibular disorders [tiab] OR Vertigo [mh] OR vertigo [tiab] OR dizziness [mh])
CINAHL	AB (“Optokinetic” OR “Optokinetic training” OR “Optokinetic response” OR “Optokinetic stimulation”) AND (“Vestibular disease” OR “Vestibular disease” OR “Vertigo” OR “dizziness”)
Web Of Science	**TOPIC** (“Optokinetic” OR “Optokinetic training” OR “Optokinetic response” OR “Optokinetic stimulation”) AND (“Vestibular disease” OR “Vestibular disease” OR “Vertigo” OR “dizziness”)
PEDro	Optokinetic AND vestibular
SCOPUS	TITLE-ABS-KEY ((“optokinetic” OR “optokinetic training” OR “optokinetic response” OR “optokinetic stimulation”) AND (“vestibular disease” OR “vestibular disorders” OR “vertigo”))

### Study selection: inclusion and exclusion criteria

To select the studies to be included in this systematic review with meta-analysis, all retrieved references were thoroughly reviewed by title and abstract. When a potential reference was identified for inclusion in the synthesis, two authors carefully reviewed that reference. Any discrepancy between the reviewers was resolved by consulting a third author, who is an expert in this topic.

The inclusion criteria were as follows: (1) experimental studies, RCTs or pilot RCTs; (2) the sample was made up of patients with vestibular and balance diseases; (3) studies that analysed the effectiveness of optokinetic stimulation; (4) the control group performed another type of intervention or no intervention; and (5) outcome measures related to subjective symptoms and objective balance evaluations. The exclusion criterion was a sample that was not entirely composed of patients with vestibular diseases.

### Data extraction

Data from selected studies were extracted and coded into a data sheet standardized Microsoft Excel file created for this review by two authors. Discrepancies were resolved by consulting a third author. The following data were collected from each of the included studies: (1) general characteristics (authorship, publication date, country and funding); (2) patient characteristics (total sample size, type of vestibular disease, number of participants in each group, age, and sex); (3) characteristics of the experimental group and the control group (type of intervention, number of sessions); and (4) data from variables (variable assessed, test employed and time-point assessment). The data used to perform our meta-analysis were the means and their standard deviations and/or differences between groups and *p* values. When a study did not provide data related to standard deviation, this was obtained from other data presented in the study, such as range, interquartile range, or standard error, as indicated by standardized statistical procedures [11, 13].

### Variables

The variables of interest were as follows: disability, which was measured using the Dizziness Handicap Inventory (DHI); postural stability, which was measured using the sensory organization test (SOT); perceived level of dizziness, which was measured using the visual analogue scale (VAS) for dizziness; and dynamic balance and risk of falls, which was measured using the timed up and go test (TUG).

### Risk of bias and methodological quality assessment

The evaluation of the risk of bias in each included study and of the quality of the evidence of the main findings was carried out by 2 authors independently. Any doubts were resolved by a third author. Initially, the methodological quality and the risk of bias of the included studies were assessed using the PEDro scale [14]. The PEDro scale is an 11-item checklist that can be scored "yes" if the criterion is met and "no" otherwise. The total score can range from 0 (very low methodological quality and high risk of bias) to 10 (excellent methodological quality and very low risk of bias), while item 1 is not used to calculate the total score because of its relationship with external validity [15]. The methodological quality of a study is considered "excellent" if it reaches a score of 9–10 points; "good" when the score ranged from 6 to 8 points; "moderate" when the score ranged from 4–5 points; and "poor" when the score was 3 or below.

Second, the quality of the evidence was analysed with the GRADE scale (Grading of Recommendations, Assessment, Development and Evaluation) and with the GRADE checklist by Meader et al. [16]. To express a level of evidence for a result, five parameters are assessed: risk of bias in the included studies, risk of publication bias in the results, inconsistency, imprecision, and indirectness. Both the risk of publication bias and heterogeneity will be explained in the statistical analysis section. The precision of the results can be high (> 10 studies or > 300 participants), moderate (10–5 studies and 300–100 participants) or low (< 5 studies and < 100 participants). Finally, evidence is considered to be indirect when scales that do not directly measure the variable of interest are used. The combination of these 5 parameters can make the quality of the evidence high, medium, low or very low. The level of evidence will be lowered for each point that is not met.

Both the evaluation of the methodological quality and the analysis of the quality of the evidence were carried out by two authors independently, and discrepancies were resolved by a third author.

### Statistical analysis

Statistical analysis was performed by 2 authors using version 4 of the Comprehensive Meta-Analysis [17]. Meta-analysis was only performed if at least two studies provided data for this meta-analysis. Following the recommendations of Cooper et al. [18], we applied a DerSimonian and Laird random-effects model to estimate the pooled effect [19]. When heterogeneity in a fixed effects model was > 50%, the random-effects model was applied. The pooled effect was calculated using Cohen’s standardized mean difference (SMD) and its 95% confidence interval (95% CI). The effect can be null (SMD 0), low (SMD 0.2–0.4), medium (SMD 0.4–0.7), and large (SMD > 0.8) [20]. The findings from each meta-analysis are shown in forest plots [21]. The risk of publication bias was assessed by analysing the symmetry of the funnel plot (if the funnel plot was asymmetric, it indicates the possibility of risk of publication bias) and the p value for Egger's test (if *p* < 0, 1 increases the risk of publication bias) [22, 23]. In addition, we used trim-and-fill estimation to calculate the adjusted event rate, taking into account a possible risk of publication bias to know whether the original findings are underestimates or overestimates [24, 25]. When the difference between the original and adjusted pooled effect was > 10%, the level of evidence was lowered by one level [26]. Heterogeneity was analysed by calculating the p value for the Higgins *Q* test (*p* < 0.1 indicates the presence of heterogeneity) and the degree of inconsistency (I^2^), which classifies heterogeneity as low (< 25%), medium (25%-50%), or large (> 50%) [27, 28].

Additionally, a subgroup analysis was planned taking into account the origin of the pathology of the included patients. The pathological subgroups were: (1) vestibular disease and (2) balance disorders without vestibular alteration.

## Results

**Fig. 1:**
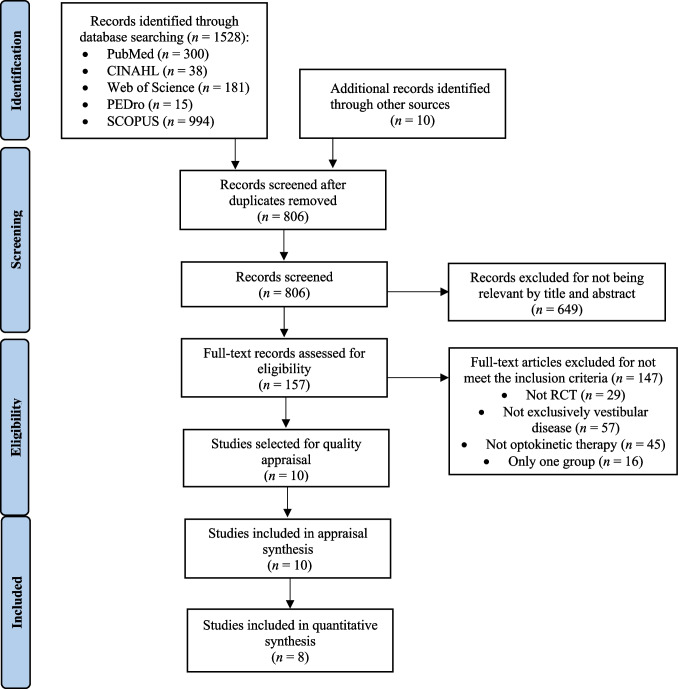
PRISMA flow diagram

After applying the eligibility criteria, a total of 10 studies [29–38] were selected (Fig. 1), including 468 patients, of whom 177 received optokinetic stimulation. Table 2 shows the main characteristics of the studies.

**Table 2:** Main characteristics of the studies included in the review

Study	Total sample size, gender and disease	OKS group					Comparison group					Outcomes
		Sample		Intervention			Sample		Intervention			Variable/test
		Ne	Age	Tt	Week	Ses/week (min)	Nc	Age	Tt	Week	Ses/week (min)	
Bunn, LM et al., 2015 (United Kingdom) Funding: Yes	12 patients (8F/4M) with cerebellar disease	6	60.2 ± 11	Balance exercise of OKS	4–8	5 (15 min)	6	58.3 ± 14.5	No intervention	NR	NR	Balance Disease severity: SARA, BAL- SARA Functional effects and impact on activity: FIM, FBS Global impact: EQ-5D, EQ-VAS, FSS, VAS
Choi, SY et al., 2021 (Korea) Funding: Yes	28 patients (16F/12M) with postural perceptual dizziness	15	75	VR + OKS	4	1 (20 min)	13	71.5	VR	4	1 (20 min)	Self-perceived impact of dizziness in daily life (DHI) VAS for dizziness Dynamic balance or risk of falls (TUG) Sensory Organization Test (SOT)
Gulcelik, GE et al., 2021 (Turkey) Funding: NR	20 patients (10F/10M) with unilateral vestibular hypofunction	10	34.3 ± 7	OKS	8	1	10	42.5 ± 12.9	Cawthorne–Cooksey protocol	8	1	Self-perceived impact of dizziness in daily life (DHI) VAS for dizziness
Loader, B et al., 2007 (Austria) Funding: NR	24 patients (15F/9M) with unilateral vestibular hypofunction with persisting disequilibrium	12	NR	Computerized OKS	3	10 (30 min)	12	NR	No intervention	NR	NR	Dynamic balance or risk of falls (TUG) Sensory organization test (SOT)
Mandour, A et al., 2022 (Egypt) Funding: No	60 patients with visual vertigo	30	NR	OKS using VR HVR (VOR X1)	4	2	30	NR	OE HVR (VOR X1)	4	NR	Self-perceived impact of dizziness in daily life (DHI)
Manso, A et al., 2016 (Brazil) Funding: Yes	40 patients (31F/9M) with chronic peripheral vestibular disease	20	45.9 (range 23–63)	OKS using ocular fixation stimulus protocol	6	2 (40 min)	20	51.85 (32–63)	Cawthorne–Cooksey protocol	6	2 (40 min)	Self-perceived impact of dizziness in daily life (DHI) VAS for dizziness
Ressiot, E et al., 2013 (France) Funding: NR	15 patients (3F/15M) wit seasickness in stage II of the Graybiel scale	7	33.1 ± 10.7	Rotational chair test OKS	10	1/2; (20 min)	8	33.4 ± 6.5	Exercises not affecting the components of seasickness	10	1/2 (20 min)	VAS for dizziness
Rossi-Izquierdo, M et al., 2011 (Spain) Funding: NR	24 patients (16F/8M) with chronic peripheral vestibular disease	12	48.8 (range 28–75)	OKS	NR	5 (15 min)	12	54.5 (range 30–82)	CDP	NR	5 (15–20 min)	Self-perceived impact of dizziness in daily life (DHI) VAS for dizziness Dynamic Balance or risk of falls (TUG) Sensory organization test (SOT)
Rossi- Izquierdo, M et al., 2017 (Spain) Funding: Yes	139 elderly patients (107F/32M) with high risk of falls without a vestibular disease	35	74 ± 5.6	OKS	2	5 (15min)	35	77.17 ± 5.72	No intervention	2	5 (15 min)	Self-perceived impact of dizziness in daily life (DHI) Dynamic balance or risk of falls (TUG) Sensory organization test (SOT)
							35	78.2 ± 6.9	CDP exercises	2	5 (15 min)	
							34	77.8 ± 6	Cawthorne–Cooksey HVR	2	2 (15 min)	
Rossi-Izquierdo, M et al., 2018 (Spain) Funding: Yes	106 elderly patients (89F/17M) with high risk of falls without a vestibular disease	30	74.3 ± 5.8	OKN	2	5 (15min)	28	76.8 ± 5.7	No intervention	2	5 (15 min)	Self-perceived impact of dizziness in daily life (DHI) FES-I Sensory organization test (SOT) Number of falls in the last 12 months
							27	76.9 ± 7.2	CDP exercises	2	5 (15 min)	
							21	76.8 ± 6.6	Cawthorne–Cooksey HVR	2	2 (15 min)	

### Assessment of the methodological quality and risk of bias of the included studies

The methodological quality of the studies included in the review was moderate according to the PEDro scale – the mean score was 4.8 points out of 10. Two studies presented good quality [32, 38], and the remaining eight had moderate quality [29–31, 33–37]. None of the studies could be blinded to the participants or the therapists, so all of them presented a risk of performance bias. Other possible identified biases were selection, detection and reporting bias. Table 3 shows the PEDro score for each study.

**Table 3:** PEDro scores of the studies included

Study	I1	I2	I3	I4	I5	I6	I7	I8	I9	I10	I11	Total	Quality
Bunn, LM et al. 2015*	Yes	Yes	Yes	Yes	No	No	No	Yes	No	Yes	Yes	6/10	Good
Choi, SY et al. 2021	Yes	Yes	No	Yes	No	No	No	Yes	No	Yes	Yes	5/10	Moderate
Gulcelik, GE et al. 2021*	Yes	Yes	No	Yes	No	No	No	Yes	No	Yes	Yes	5/10	Moderate
Loader, B et al. 2007*	Yes	Yes	No	No	No	No	No	Yes	No	Yes	Yes	4/10	Moderate
Mandour, AES et al. 2022*	Yes	Yes	Yes	Yes	No	No	No	No	No	Yes	Yes	5/10	Moderate
Manso, A et al. 2016*	No	Yes	No	Yes	No	No	Yes	No	No	Yes	Yes	4/10	Moderate
Ressiot, E et al. 2013	Yes	Yes	No	Yes	No	No	Yes	Yes	No	Yes	Yes	6/10	Good
Rossi-Izquierdo, M et al. 2011*	Yes	Yes	No	Yes	No	No	No	No	No	Yes	Yes	4/10	Moderate
Rossi-Izquierdo, M et al. 2017*	No	Yes	No	Yes	No	No	No	Yes	No	Yes	Yes	5/10	Moderate
Rossi-Izquierdo, M et al. 2018*	No	Yes	No	Yes	No	No	No	Yes	No	Yes	No	4/10	Moderate

*I1* eligibility criteria, *I2* randomized distribution, *I3* allocation concealment, *I4* comparability at baseline, *I5* blinded subjects, *I6* blinded therapists, *I7* blinded assessors, *I8* adequate monitoring, *I9* intention-to-treat analysis, *I10* between-groups comparison, *I11* point estimation and variability, Note: Item 1 does not contribute to the final score. Note: *Score confirmed in PEDro webpage

### Dizziness handicap inventory (DHI)

**Fig. 2:**
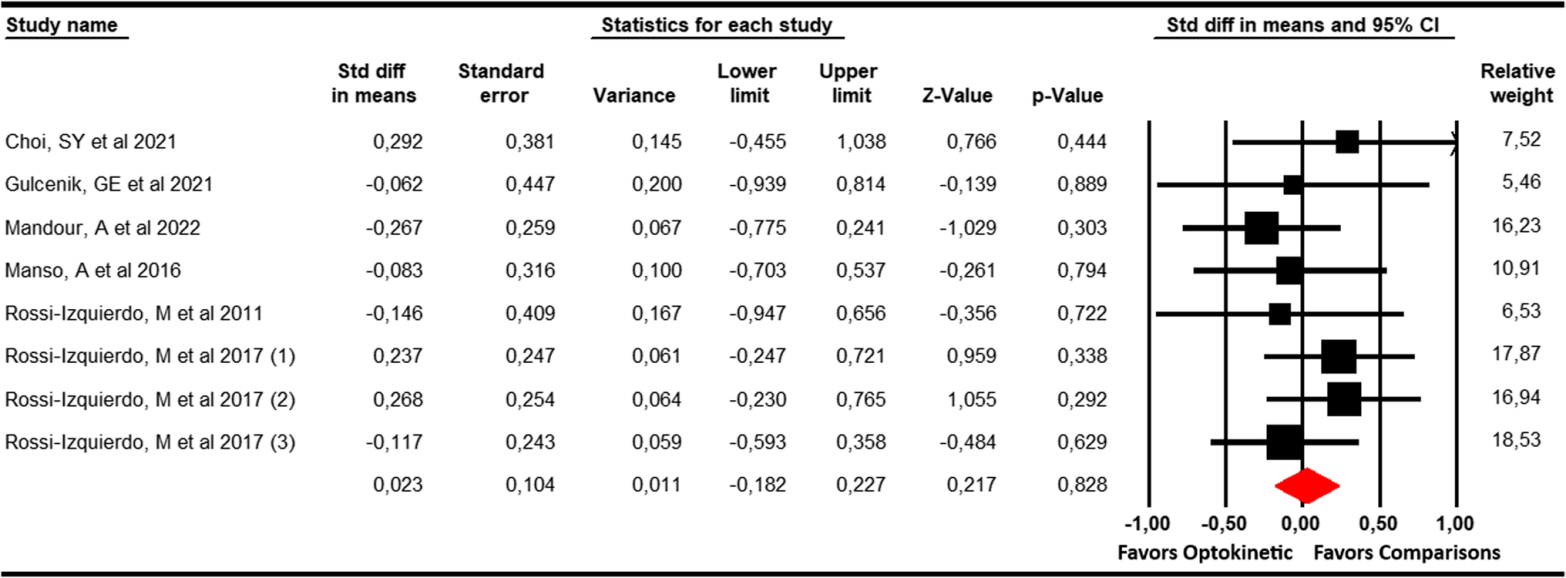
Forest plot showing the pooled effects of Optokinetic Stimulation compared with other interventions on the Dizziness Handicap Inventory scores

Six studies [29, 30, 33, 34, 36, 37] with 8 independent comparisons providing data from 369 patients (46.1 per study) were included in this meta-analysis. Our results did not show statistically significant differences (SMD = 0.02; 95% CI -0.18 to 0.23; *p* = 0.83) between optokinetic therapy and other therapies (Table 4, Fig. 2). No heterogeneity (*I*^2^ = 0%; *Q* = 4.1; *df* = 7; *p* = 0.77) and no risk of publication bias (Egger *p* = 0.86) were observed.

**Table 4:** Main findings in meta-analyses and GRADE assessment

Variable	Summary of findings											Quality of the evidence (GRADE assessment)					
	Effect size						Heterogeneity		Publication bias risk								
	K	N	Ns	SMD	95% CI	*p*	Q (df)	I^2^ *(p)*	Egger *p*	Trim and Fill		Risk of bias	Incons	Indir	Impr	Publication bias	Quality of evidence
										Adj SMD	% Var						
DHI	8	369	46.1	0.02	− 0.18 to 0.23	0.83	4.1 (7)	0% (0.77)	0.86	0.02	0	Medium	No	No	Yes	No	Low
VAS for dizziness	4	103	25.8	0.16	− 1.25 to 1.58	0.82	3.8 (3)	20.1% (0.29)	0.73	0.23	0	Medium	Yes	No	Yes	Yes	Very low
Timed up and go test	4	225	57.3	− 1.13	− 2 to − 0.2	0.009	7.8 (3)	61.4% (0.05	0.07	− 1.5	32	Medium	Yes	No	Yes	Yes	Very low
Sensory organization test (SOT)	6	225	37.5	− 0.7	− 1.21 to − 0.19	0.007	5.6 (5)	13% (0.35)	0.27	− 0.7	0	Medium	Yes	No	Yes	No	Very low

*K* number of comparisons, *N* sample size, *Ns* participants per study, *SMD* standardized mean difference, *95% CI* 95% Confidence interval, *p* p-value, *Q* Chi-squared test, *df* degree of freedom, *I*^2^ degree of inconsistency, *Adj SMD* adjusted effect, *% var* % of variation; Incons, inconsistency; Indir, indirect evidence; Impr, Imprecision; Public, publication, *NP* not possible to calculate

To perform this subgroup analysis, we identified 3 studies with 3 independent comparisons with patients with vestibular disorders [29, 34, 37]; and 3 studies with 5 independent comparisons that provided data from patients with balance disorders [30, 33, 36]. Subgroup analyses did not show statistically significant differences favouring OKS in reducing DHI in patients with balance disorders (SMD = 0.58; 95% CI − 0.18 to 0.3; *p* = 0.63; *I*^2^ = 0%; *Q* = 3.7; *df* = 4; *p* = 0.49) and vestibular disorders (SMD = − 0.1; 95% CI − 0.52 to 0.33; *p* = 0.66; *I*^2^ = 0%; *Q* = 0.1; *df* = 3; *p* = 0.99).

### Visual analogue scale (VAS) for dizziness

**Fig. 3:**
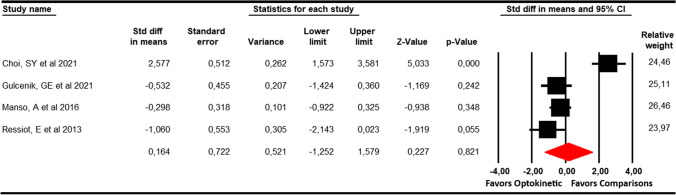
Forest plot showing the pooled effects of Optokinetic Stimulation compared to other interventions for the improvement of Visual Analogue Scale for dizziness

Four studies [33, 34, 37, 38] with 4 independent comparisons reporting data from 103 patients (25.8 per study) were used to perform this meta-analysis. No statistically significant differences (SMD = 0.16; 95% CI −1.25 to 1.58;  *p = 0.82*) were found between optokinetic therapy and other interventions (Table 4, Fig. 3). Although a risk of publication bias was present (Egger *p* = 0.73; adjusted SMD with trim-and-fill estimation = 0.23), it did not change the original effect. The level of heterogeneity was low (*I*^2^ = 20.1%; *Q* = 3.8; *df* = 3; *p* = 0.29).

For subgroup analyses, 2 studies with 2 independent comparisons reported data from patients with vestibular diseases [34, 37]; and others 2 studies with 2 independent comparisons for patients with balance disorders [33, 38]. Our subgroup analyses reported that OKS is effective in reducing VAS for dizziness in patients with balance disorders (SMD = 0.9; 95% CI 0.16 to 1.63; *p* = 0.017; *I*^2^ = 79.1%; *Q* = 21.1; *df* = 1; *p* < 0.001), but no in patients with vestibular diseases (SMD = − 0.38; 95% CI − 0.89 to 0.14; *p* = 0.15: *I*^2^ = 0%; *Q* = 0.17; *df* = 1; *p* = 0.68).

### Timed up and go (TUG) test

**Fig. 4:**
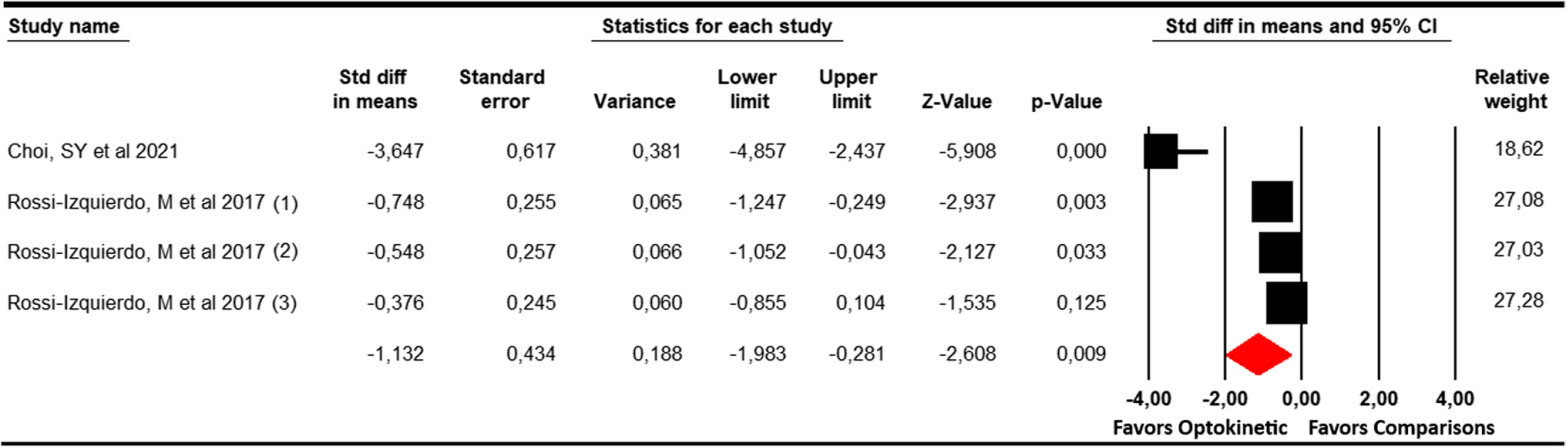
Forest plot showing the pooled effects of Optokinetic Stimulation compared to other interventions on the Timed Up and Go Test

In this meta-analysis, 2 studies [30, 33] with 4 independent comparisons that provided data from 225 patients with balance disorders without vestibular disease (57.3 per study) were included. Our findings showed a large effect (SMD = − 1.13; 95% CI − 2 to − 0.28; *p* = 0.009) favouring optokinetic therapy (Table 4, Fig. 4). Trim-and-fill estimation showed a possible risk of publication bias and reported an adjusted effect (adjusted SMD = − 1.5) major than the original effect, taking into account this risk of publication bias. This evidence indicates that the original effect can be underestimated by publication bias. However, the level of heterogeneity in a random-effects model was large (*I*^2^ = 61.4%; *Q* = 7.8; *df* = 3; *p* = 0.05).

### Sensory organization test (SOT)

**Fig. 5:**
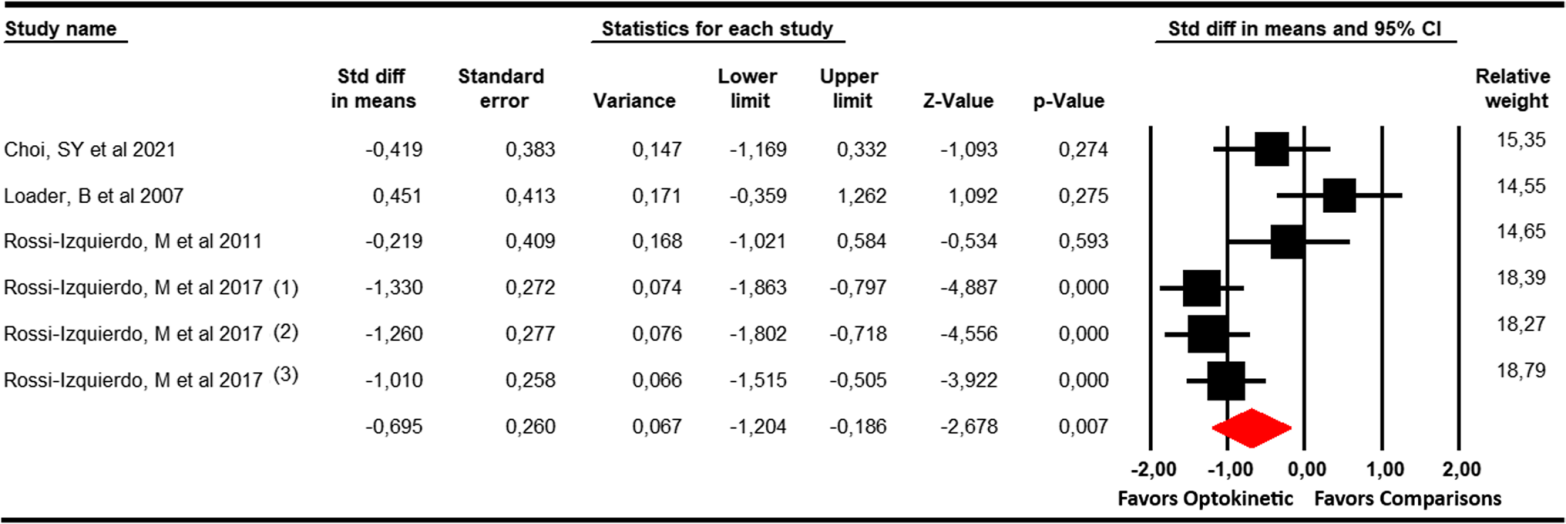
Forest plot showing the pooled effects of Optokinetic Stimulation compared to other interventions on the Sensory Organization Test (SOT)

Four studies [29, 30, 33, 35] with 6 independent comparisons providing data from 225 patients (37.5 per study) were included in this meta-analysis. Our results showed that a medium–large effect (SMD = − 0.7; 95% CI − 1.21 to − 0.19; *p* = 0.007) favours optokinetic therapy (Table 4, Fig. 5) in improving composite measurement. Heterogeneity was low (*I*^2^ = 13%; *Q* = 5.6; *df* = 5; *p* = 0.35), and the risk of publication bias was not present.

For subgroup analyses, 2 studies with 2 independent comparisons reported data from patients with vestibular diseases [29, 35]; and others 2 studies with 2 independent comparisons for patients with balance disorders [30, 33]. Our subgroup analyses reported that OKS is effective in improving SOT in patients with balance disorders (SMD = − 1.07; 95% CI − 1.41 to − 0.72; *p* < 0.001; *I*^2^ = 6.6%; *Q* = 3.2; *df* = 3; *p* = 0.36), but no in patients with vestibular diseases (SMD = 0.11; 95% CI − 0.52 to 0.75; *p* = 0.73; *I*^2^ = 6.9%; *Q* = 1.1; *df* = 1; *p* = 0.29).

### Qualitative synthesis

Additionally, two studies provided data that could not be integrated with that obtained by the rest of the studies. Rossi-Izquierdo 2018 et al. [31], found a significant reduction in the average number of falls at 12 months of follow-up in the OKN group, which went from 17.07 to 4.43 (*p* = 0.011) compared to the control group that showed a reduction from 3.36 to 2.61, which was statistically non-significant (*p* = 0.166). In the study by Bunn 2015 et al. [32], the OKN group showed a reduction in the Sway of the Centre of Pressure that was greater than the control group in moving visual scenery (MVS) situations. These results provide evidence of an improvement in balance in dynamic conditions and a reduction in the number of falls in subjects undergoing therapy with OKN.

## Discussion

Our review aimed to locate and analyse the best evidence on the effectiveness of OKS for the improvement of symptoms and function in subjects with vestibular and balance disorders. Our findings show limited and generally low-quality evidence indicating that optokinetics had a large effect on improving dynamic balance (as measured by the TUG and CDP) and no effect on subjective measures such as the DHI and VAS for vertigo.

Our review found low-quality evidence that optokinetics have no effect on improving disability due to dizziness (as measured by the DHI) when compared to other interventions, contrary to the investigators' initial hypothesis. Some previous studies have shown that vestibular rehabilitation therapy and corticosteroids can be a good combination for improving DHI in vestibular neuritis [39], propranolol can be effective in patients with vestibular migraine [40], and vestibular rehabilitation can be effective in patients with acute vestibular disorder [41] and in Meniere’s disease [42]. Therefore, and in the absence of more robust evidence, vestibular rehabilitation and different pharmacological measures could be better measures to improve subjective symptoms. Virtual reality-assisted therapy has been shown to have additional benefits in patients with vestibular disorders compared with conventional vestibular physical therapy for the improvement of the DHI total scores and its subscales [43]. It is important to note that most of the comparisons in our meta-analysis consisted of active therapy that has been shown to be effective in improving subjective symptoms, so our findings can be interpreted as meaning that OKS may have a similar but not superior effect to these types of interventions, such as Cawthorne–Cooksey exercises or rehabilitation with CDP.

Additionally, in relation to subjective symptoms, another variable used in the trials included in the review was the VAS of vertigo. With very low quality of evidence, our findings showed that there was no effect of OKS when compared with other interventions or no intervention. In patients with acute vertigo, the use of benzodiazepines and antihistamines has shown immediate beneficial effects on the VAS for vertigo [44]. In subjects with peripheral vestibular dysfunction, the use of virtual reality as a vestibular rehabilitative intervention was able to improve VAS scores [45].

Regarding objective measurements, very low-quality evidence from four comparisons found a large effect of OKS for the improvement of dynamic balance (measured with the TUG test). The evidence on improvement of this variable in patients with vestibular and balance disorders is poorer and derives from isolated clinical trials, where improvement was observed in all arms of the trial. An improvement in the TUG test has been found with home rehabilitation programs in patients with chronic vertigo due to peripheral vestibular impairments [46] through the use of vestibular rehabilitation in older people with chronic dizziness [47], although other treatments, such as the use of rehabilitation with CDP, seem to have no effect on improving stability in older patients with instability [48].

Regarding SOT, few interventions have been successfully tested to improve composite measurement. Vestibular rehabilitation seems to improve SOT in patients with multiple sclerosis [49], but the results of the treatment of peripheral or central vestibular disorders using virtual reality-assisted therapy do not seem to provide a significant effect [43]. In our study, the effect of OKS on improving SOT was moderate when compared with other interventions but with a very low quality of evidence.

This article has several limitations. First, the evidence found is scarce, with few randomized clinical trials that adopted a very heterogeneous methodology, which makes it difficult to establish specific recommendations. Therefore, the quality of the evidence is low or very low in the meta-analyses, and partially supported recommendations for practice can be made. Additionally, recommendations for new research can also be made. Despite the above limitations, to the best of our knowledge, this is the first review that has been carried out on the topic. These findings can improve the general approach and be a starting point for new and more oriented research.

## Conclusions

In view of our results, it can be concluded that there is low and very low evidence that OKS is not better than other interventions or no intervention for the improvement of disability due to dizziness or the subjective perception of dizziness, respectively, in subjects with vestibular and balance disorders.

There is very low evidence indicating that OKS has a large beneficial effect on dynamic balance (measured with the TUG test) in subjects with balance disorders when compared with other interventions or no intervention. We also found very low-quality evidence indicating that OKS has a medium-sized beneficial effect on balance (as measured by the SOT) in subjects with vestibular and balance disorders when compared with other interventions or no intervention.

In reference to the effect on different pathological conditions, the OKS showed a significant effect for the decrease in the intensity of dizziness and for the improvement of dynamic balance measured with TUG and SOT in patients with balance disorders of diverse origins, but no significant effect was found in subjects with vestibular disease for none of the variables analysed.

OKS offers promising results in improving objective measurements of balance, although the methodological heterogeneity of the trials included in the review does not allow more precise conclusions to be drawn. Therefore, new research with better standardized methodology is needed to analyse the real effect of optokinetic stimulation in different groups of patients.

## Data Availability

The data of this study is available under reasonable request to the corresponding author.

## References

[1] Agrawal Y, Ward BK, Minor LB (2013) Vestibular dysfunction: prevalence, impact and need for targeted treatment. J Vestib Res 23:113. 10.3233/VES-130498 24177344 10.3233/VES-130498 PMC4069154

[2] Curry SD, Carotenuto A, Huang Y et al (2023) Older adults with vestibular disorders and hip fractures have high rates of meclizine use. Otol Neurotol 44:E178–E183. 10.1097/MAO.0000000000003792 36728629 10.1097/MAO.0000000000003792

[3] Bronstein AM (2019) A conceptual model of the visual control of posture. Prog Brain Res 248:285–302. 10.1016/BS.PBR.2019.04.023 31239139 10.1016/BS.PBR.2019.04.023

[4] Liu KPY, Hanly J, Fahey P et al (2019) A systematic review and meta-analysis of rehabilitative interventions for unilateral spatial neglect and hemianopia poststroke from 2006 through 2016. Arch Phys Med Rehabil 100:956–979. 10.1016/j.apmr.2018.05.037 31030733 10.1016/j.apmr.2018.05.037

[5] Pavlou M (2010) The use of optokinetic stimulation in vestibular rehabilitation. J Neurol Phys Ther 34:105–110. 10.1097/NPT.0B013E3181DDE6BF 20588097 10.1097/NPT.0B013E3181DDE6BF

[6] Dieterich M, Bucher SF, Seelos KC, Brandt T (1998) Horizontal or vertical optokinetic stimulation activates visual motion-sensitive, ocular motor and vestibular cortex areas with right hemispheric dominance. An fMRI study. Brain 121:1479–1495. 10.1093/BRAIN/121.8.1479 9712010 10.1093/BRAIN/121.8.1479

[7] Pavlou M, Quinn C, Murray K et al (2011) The effect of repeated visual motion stimuli on visual dependence and postural control in normal subjects. Gait Posture 33:113–118. 10.1016/j.gaitpost.2010.10.085 21144753 10.1016/j.gaitpost.2010.10.085

[8] Pavlou M, Kanegaonkar RG, Swapp D et al (2012) The effect of virtual reality on visual vertigo symptoms in patients with peripheral vestibular dysfunction: a pilot study. J Vestib Res 22:273–281. 10.3233/VES-120462 23302708 10.3233/VES-120462

[9] Vitte E, Smont A, Berthoz A (1994) Repeated optokinetic stimulation in conditions of active standing facilitates recovery from vestibular deficits. Springer-Verlag 10.1007/BF00232446 7895790

[10] Page MJ, McKenzie JE, Bossuyt PM et al (2021) The PRISMA 2020 statement: an updated guideline for reporting systematic reviews. BMJ 372:n71. 10.1136/bmj.n71 33782057 10.1136/bmj.n71 PMC8005924

[11] Higgins J, Green S (2011) Cochrane handbook for systematic reviews of interventions. London

[12] Eriksen MB, Frandsen TF (2018) The impact of patient, intervention, comparison, outcome (PICO) as a search strategy tool on literature search quality: a systematic review. J Med Lib Assoc. 10.5195/JMLA.2018.345 10.5195/JMLA.2018.345 PMC6148624 30271283

[13] Hozo SP, Djulbegovic B, Hozo I (2005) Estimating the mean and variance from the median, range, and the size of a sample. BMC Med Res Methodol 5:13. 10.1186/1471-2288-5-13 15840177 10.1186/1471-2288-5-13 PMC1097734

[14] Moseley AM, Herbert RD, Sherrington C, Maher CG (2002) Evidence for physiotherapy practice: a survey of the Physiotherapy Evidence Database (PEDro). Australian Journal of Physiotherapy 48:43–49. 10.1016/S0004-9514(14)60281-6 11869164 10.1016/S0004-9514(14)60281-6

[15] Maher CG, Sherrington C, Herbert RD et al (2003) Reliability of the PEDro scale for rating quality of randomized controlled trials. Phys Ther 83:713–721 12882612 10.1093/ptj/83.8.713

[16] Meader N, King K, Llewellyn A et al (2014) A checklist designed to aid consistency and reproducibility of GRADE assessments: development and pilot validation. Syst Rev 3:82. 10.1186/2046-4053-3-82 25056145 10.1186/2046-4053-3-82 PMC4124503

[17] Borenstein M, Hedges L, Higgins J, Rothstein H (2020) Comprehensive meta-analysis software version 4

[18] Cooper H, Hedges LV, Valentine JC (2009) The handbook of research synthesis and meta-analysis, 2nd edn. Russell Sage Foundation, New York

[19] DerSimonian R, Laird N (1986) Meta-analysis in clinical trials. Control Clin Trials 7:177–188. 10.1016/0197-2456(86)90046-2 3802833 10.1016/0197-2456(86)90046-2

[20] Cohen J (1977) Statistical power analysis for the behavioral sciences. Academic Press, New York

[21] Rücker G, Schwarzer G (2020) Beyond the forest plot: the drapery plot. Res Synth Methods. 10.1002/jrsm.1410 32336044 10.1002/jrsm.1410

[22] Sterne JAC, Egger M (2001) Funnel plots for detecting bias in meta-analysis: guidelines on choice of axis. J Clin Epidemiol 54:1046–1055. 10.1016/S0895-4356(01)00377-8 11576817 10.1016/S0895-4356(01)00377-8

[23] Egger M, Smith GD, Schneider M, Minder C (1997) Bias in meta-analysis detected by a simple, graphical test measures of funnel plot asymmetry. BMJ 315:629–634. 10.1136/bmj.315.7109.629 9310563 10.1136/bmj.315.7109.629 PMC2127453

[24] Duval S, Tweedie R (2000) Trim and fill: a simple funnel-plot-based method of testing and adjusting for publication bias in meta-analysis. Biometrics 56:455–463. 10.1111/j.0006-341X.2000.00455.x 10877304 10.1111/j.0006-341X.2000.00455.x

[25] Shi L, Lin L, Omboni S (2019) The trim-and-fill method for publication bias: practical guidelines and recommendations based on a large database of meta-analyses. Medicine. 10.1097/MD.0000000000015987 31169736 10.1097/MD.0000000000015987 PMC6571372

[26] Rothman KJ, Greenland S, Lash TL (2008) Modern epidemiology. Lippincott Williams & Wilkins, Philadelphia

[27] Higgins J, Thompson S, Deeks J, Altman D (2003) Measuring inconsistency in meta-analyses. BMJ 327:557–560. 10.1136/bmj.327.7414.557 12958120 10.1136/bmj.327.7414.557 PMC192859

[28] Higgins J, Thompson S, Deeks J, Altman D (2002) Statistical heterogeneity in systematic reviews of clinical trials: a critical appraisal of guidelines and practice. J Health Serv Res Policy 7:51–61. 10.1258/1355819021927674 11822262 10.1258/1355819021927674

[29] Rossi-Izquierdo M, Santos-Pérez S, Soto-Varela A (2011) What is the most effective vestibular rehabilitation technique in patients with unilateral peripheral vestibular disorders? Eur Arch Otorhinolaryngol 268:1569–1574. 10.1007/s00405-011-1532-z 21340557 10.1007/s00405-011-1532-z

[30] Rossi-Izquierdo M, Gayoso-Diz P, Santos-Pérez S et al (2017) Short-term effectiveness of vestibular rehabilitation in elderly patients with postural instability: a randomized clinical trial. Eur Arch Otorhinolaryngol 274:2395–2403. 10.1007/s00405-017-4472-4 28251319 10.1007/s00405-017-4472-4

[31] Rossi-Izquierdo M, Gayoso-Diz P, Santos-Pérez S et al (2018) Vestibular rehabilitation in elderly patients with postural instability: reducing the number of falls—a randomized clinical trial. Aging Clin Exp Res 30:1353–1361. 10.1007/s40520-018-1003-0 30008159 10.1007/s40520-018-1003-0

[32] Bunn LM, Marsden JF, Giunti P, Day BL (2015) Training balance with opto-kinetic stimuli in the home: a randomized controlled feasibility study in people with pure cerebellar disease. Clin Rehabil 29:143–153. 10.1177/0269215514539336 25082955 10.1177/0269215514539336

[33] Choi SY, Choi JH, Oh EH et al (2021) Effect of vestibular exercise and optokinetic stimulation using virtual reality in persistent postural-perceptual dizziness. Sci Rep. 10.1038/s41598-021-93940-z 34262120 10.1038/s41598-021-93940-z PMC8280184

[34] Gulcelik EG, Tarakci D, Gedik Soyuyuce O et al (2021) Research on the effects of a web-based system with oculomotor and optokinetic stimuli on vestibular rehabilitation. Am J Phys Med Rehabil 100:555–562. 10.1097/PHM.0000000000001584 32889859 10.1097/PHM.0000000000001584

[35] Loader B, Gruther W, Mueller CA, et al (2007) Improved postural control after computerized optokinetic therapy based on stochastic visual stimulation in patients with vestibular dysfunction. J Vesti Res. 131–136 18413906

[36] Mandour AE et al (2022) Virtual reality versus optokinetic stimulation in visual vertigo rehabilitation. Eur Arch Otorhinolaryngol 279:1609–1614 34611745 10.1007/s00405-021-07091-y

[37] Manso A, Ganança MM, Caovilla HH (2016) Vestibular rehabilitation with visual stimuli in peripheral vestibular disorders. Braz J Otorhinolaryngol 82:232–241 26832632 10.1016/j.bjorl.2015.05.019 PMC9449018

[38] Ressiot E, Dolz M, Bonne L, Marianowski R (2013) Prospective study on the efficacy of optokinetic training in the treatment of seasickness. Eur Ann Otorhinolaryngol Head Neck Dis 130:263–268. 10.1016/j.anorl.2012.03.009 23562228 10.1016/j.anorl.2012.03.009

[39] Badriyah Hidayati H, Aqilah Nur Imania H, Sella Octaviana D et al (2022) Vestibular rehabilitation therapy and corticosteroids for vestibular neuritis: a systematic review and meta-analysis of randomized controlled trials. Medicina. 10.3390/medicina58091221 10.3390/medicina58091221 PMC9506214 36143898

[40] Yiannakis C, Hamilton L, Slim M, Kontorinis G (2023) A systematic review and meta-analysis of prophylactic medication of vestibular migraine. J Laryngol Otol 137:953–961. 10.1017/S0022215122001979 36200521 10.1017/S0022215122001979

[41] Kamo T, Ogihara H, Azami M et al (2023) Effects of early vestibular rehabilitation in patients with acute vestibular disorder: a systematic review and meta-analysis. Otol Neurotol 44:e641–e647. 10.1097/MAO.0000000000004006 37641214 10.1097/MAO.0000000000004006

[42] Rezaeian A, Abtahi H, Moradi M, Farajzadegan Z (2023) The effect of vestibular rehabilitation in Meniere’s disease: a systematic review and meta-analysis of clinical trials. Eur Arch Otorhinolaryngol 280:3967–3975. 10.1007/S00405-023-08066-X/METRICS 37341761 10.1007/S00405-023-08066-X/METRICS

[43] Chu HY, Song N, Zhou ZR et al (2023) Can virtual reality-assisted therapy offer additional benefits to patients with vestibular disorders compared with conventional vestibular physical therapy? A meta-analysis. Arch Phys Med Rehabil 104:490–501. 10.1016/j.apmr.2022.08.972 36265531 10.1016/j.apmr.2022.08.972

[44] Hunter BR, Wang AZ, Bucca AW et al (2022) Efficacy of benzodiazepines or antihistamines for patients with acute vertigo: a systematic review and meta-analysis. JAMA Neurol 79:846. 10.1001/JAMANEUROL.2022.1858 35849408 10.1001/JAMANEUROL.2022.1858 PMC9295021

[45] Hazzaa NM, Manzour AF, Yahia E, Mohamed Galal E (2023) Effectiveness of virtual reality-based programs as vestibular rehabilitative therapy in peripheral vestibular dysfunction: a meta-analysis. Eur Arch Otorhinolaryngol 280:3075–3086. 10.1007/S00405-023-07911-3/TABLES/5 36947249 10.1007/S00405-023-07911-3/TABLES/5 PMC10220119

[46] Cohen HS, Kimball KT (2004) Decreased ataxia and improved balance after vestibular rehabilitation. Otolaryngol Head Neck Surg 130:418–443. 10.1016/j.otohns.2003.12.020 15100637 10.1016/j.otohns.2003.12.020

[47] Ricci NA, Aratani MC, Caovilla HH, Ganança FF (2016) Effects of vestibular rehabilitation on balance control in older people with chronic dizziness: a randomized clinical trial. Am J Phys Med Rehabil 95:256–269. 10.1097/PHM.0000000000000370 26368833 10.1097/PHM.0000000000000370

[48] Soto-Varela A, Rossi-Izquierdo M, Del-Río-valeiras M et al (2020) Vestibular rehabilitation using posturographic system in elderly patients with postural instability: can the number of sessions be reduced? Clin Interv Aging 15:991–1001. 10.2147/CIA.S263302 32617000 10.2147/CIA.S263302 PMC7326163

[49] Lotfi Y, Farahani A, Azimiyan M et al (2021) Comparison of efficacy of vestibular rehabilitation and noisy galvanic vestibular stimulation to improve dizziness and balance in patients with multiple sclerosis. J Vestib Res 31:541–551. 10.3233/VES-201609 33967075 10.3233/VES-201609

